# Bromido(2-{1-[2-(morpholin-4-yl)ethyl­imino]­eth­yl}phenolato)copper(II)

**DOI:** 10.1107/S160053681002670X

**Published:** 2010-07-10

**Authors:** Xiao-Fan Zhao, Fang Li

**Affiliations:** a College of Chemistry & Chemical Engineering, Shaoxing University, Shaoxing 312000, People’s Republic of China

## Abstract

In the title complex, [CuBr(C_14_H_19_N_2_O_2_)], the Cu^II^ atom is coordinated by one phenolate O, one imine N and one amine N atom of the tridentate Schiff base ligand and by one bromide ion, resulting in a distorted CuBrN_2_O square-planar geometry, with the N atoms in a *cis* arrangement. The morpholine ring adopts a chair conformation.

## Related literature

For background to Schiff base complexes and a related structure, see: Zhao (2008 ▶). For similar copper(II) complexes with Schiff bases, see: Zhu *et al.* (2005 ▶); Ni *et al.* (2005 ▶); Zhu (2010 ▶); Suleiman Gwaram *et al.* (2010 ▶).
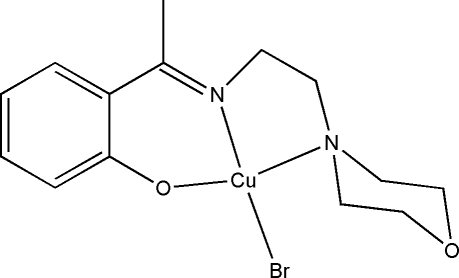


## Experimental

### 

#### Crystal data

[CuBr(C_14_H_19_N_2_O_2_)]
*M* *_r_* = 390.76
Monoclinic, 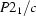

*a* = 10.808 (2) Å
*b* = 17.152 (3) Å
*c* = 8.107 (2) Å
β = 90.059 (1)°
*V* = 1502.9 (5) Å^3^
*Z* = 4
Mo *K*α radiation
μ = 4.11 mm^−1^
*T* = 298 K
0.32 × 0.30 × 0.30 mm

#### Data collection

Bruker SMART CCD diffractometer
Absorption correction: multi-scan (*SADABS*; Sheldrick, 1996 ▶) *T*_min_ = 0.353, *T*_max_ = 0.372
9814 measured reflections
3211 independent reflections
2506 reflections with *I* > 2σ(*I*)
*R*_int_ = 0.041

#### Refinement

*R*[*F* ^2^ > 2σ(*F* ^2^)] = 0.074
*wR*(*F* ^2^) = 0.171
*S* = 1.13
3211 reflections
182 parameters
H-atom parameters constrained
Δρ_max_ = 1.06 e Å^−3^
Δρ_min_ = −1.06 e Å^−3^

### 

Data collection: *SMART* (Bruker, 1998 ▶); cell refinement: *SAINT* (Bruker, 1998 ▶); data reduction: *SAINT*; program(s) used to solve structure: *SHELXS97* (Sheldrick, 2008 ▶); program(s) used to refine structure: *SHELXL97* (Sheldrick, 2008 ▶); molecular graphics: *SHELXTL* (Sheldrick, 2008 ▶); software used to prepare material for publication: *SHELXTL*.

## Supplementary Material

Crystal structure: contains datablocks global, I. DOI: 10.1107/S160053681002670X/hb5543sup1.cif

Structure factors: contains datablocks I. DOI: 10.1107/S160053681002670X/hb5543Isup2.hkl

Additional supplementary materials:  crystallographic information; 3D view; checkCIF report

## Figures and Tables

**Table:** 

Cu1—O1	1.877 (6)
Cu1—N1	1.917 (7)
Cu1—N2	2.095 (6)
Cu1—Br1	2.4006 (14)

**Table:** 

O1—Cu1—N1	91.1 (3)
O1—Cu1—N2	161.7 (3)
N1—Cu1—N2	87.5 (2)
O1—Cu1—Br1	92.2 (2)
N1—Cu1—Br1	157.9 (2)
N2—Cu1—Br1	95.99 (16)

**Fig. 1.**
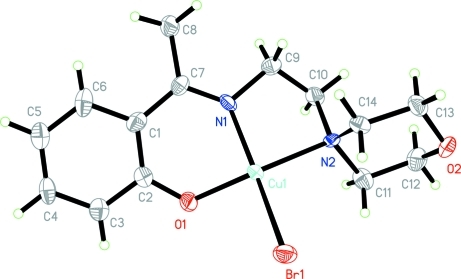
The structure of (I), showing 30% displacement ellipsoids (arbitrary spheres for the H atoms).

## supplementary crystallographic information

### Comment

As part of our ongoing studies of Schiff base complexes (e.g. Zhao, 2008), the title mononuclear copper(II) complex, (I), is reported here.

In the title complex, the Cu atom is four-coordinated by one phenolate O, one imine N, and one amine N atoms of 2-[1-(2-morpholin-4-ylethylimino)ethyl]phenolate, and by one bromide atom, forming a square planar geometry (Fig. 1). The bond lengths (Table 1) in the square planar coordination are comparable with those reported in similar copper structures with Schiff bases (Zhu *et al.*, 2005; Ni *et al.*, 2005; Zhu, 2010; Suleiman Gwaram *et al.*, 2010).

### Experimental

1-(2-Hydroxyphenyl)ethanone (1 mmol, 136 mg), 2-morpholin-4-ylethylamine (1 mmol, 130 mg), and copper(II) bromide (1 mmol, 223 mg) were dissolved in methanol (80 ml). The mixture was stirred at room temperature for 1 h to give a blue solution. The resulting solution was kept in air for a week, and blue blocks of (I) were formed.

### Refinement

H atoms were placed in idealized positions and constrained to ride on their parent atoms, with C—H distances in the range 0.93–0.97 Å, and with *U*_iso_(H) = 1.2 or 1.5*U*_eq_(C).

### Crystal data

**Table:**

[CuBr(C_14_H_19_N_2_O_2_)]	*F*(000) = 788
*M**_r_* = 390.76	*D*_x_ = 1.727 Mg m^−^^3^
Monoclinic, *P*2_1_/*c*	Mo *K*α radiation, λ = 0.71073 Å
Hall symbol: -P 2ybc	Cell parameters from 3491 reflections
*a* = 10.808 (2) Å	θ = 2.7–26.4°
*b* = 17.152 (3) Å	µ = 4.11 mm^−^^1^
*c* = 8.107 (2) Å	*T* = 298 K
β = 90.059 (1)°	Block, blue
*V* = 1502.9 (5) Å^3^	0.32 × 0.30 × 0.30 mm
*Z* = 4	

### Data collection

**Table:**

Bruker SMART CCD diffractometer	3211 independent reflections
Radiation source: fine-focus sealed tube	2506 reflections with *I* > 2σ(*I*)
graphite	*R*_int_ = 0.041
ω scans	θ_max_ = 27.0°, θ_min_ = 2.2°
Absorption correction: multi-scan (*SADABS*; Sheldrick, 1996)	*h* = −13→12
*T*_min_ = 0.353, *T*_max_ = 0.372	*k* = −21→21
9814 measured reflections	*l* = −10→10

### Refinement

**Table:**

Refinement on *F*^2^	Primary atom site location: structure-invariant direct methods
Least-squares matrix: full	Secondary atom site location: difference Fourier map
*R*[*F*^2^ > 2σ(*F*^2^)] = 0.074	Hydrogen site location: inferred from neighbouring sites
*wR*(*F*^2^) = 0.171	H-atom parameters constrained
*S* = 1.13	*w* = 1/[σ^2^(*F*_o_^2^) + (0.0288*P*)^2^ + 17.4414*P*] where *P* = (*F*_o_^2^ + 2*F*_c_^2^)/3
3211 reflections	(Δ/σ)_max_ < 0.001
182 parameters	Δρ_max_ = 1.06 e Å^−^^3^
0 restraints	Δρ_min_ = −1.06 e Å^−^^3^

### Special details

**Table:**

Geometry. All e.s.d.'s (except the e.s.d. in the dihedral angle between two l.s. planes) are estimated using the full covariance matrix. The cell e.s.d.'s are taken into account individually in the estimation of e.s.d.'s in distances, angles and torsion angles; correlations between e.s.d.'s in cell parameters are only used when they are defined by crystal symmetry. An approximate (isotropic) treatment of cell e.s.d.'s is used for estimating e.s.d.'s involving l.s. planes.
Refinement. Refinement of *F*^2^ against ALL reflections. The weighted *R*-factor *wR* and goodness of fit *S* are based on *F*^2^, conventional *R*-factors *R* are based on *F*, with *F* set to zero for negative *F*^2^. The threshold expression of *F*^2^ > σ(*F*^2^) is used only for calculating *R*-factors(gt) *etc*. and is not relevant to the choice of reflections for refinement. *R*-factors based on *F*^2^ are statistically about twice as large as those based on *F*, and *R*- factors based on ALL data will be even larger.

### Fractional atomic coordinates and isotropic or equivalent isotropic displacement parameters (Å^2^)

**Table:**

	*x*	*y*	*z*	*U*_iso_*/*U*_eq_	
Cu1	0.98896 (9)	0.13655 (6)	0.00058 (11)	0.0314 (3)	
Br1	1.13378 (9)	0.10662 (6)	−0.21461 (11)	0.0482 (3)	
N1	0.8594 (6)	0.1190 (4)	0.1576 (8)	0.0364 (16)	
N2	1.1147 (5)	0.1243 (4)	0.1957 (7)	0.0268 (13)	
O1	0.8763 (6)	0.1804 (4)	−0.1494 (8)	0.0513 (17)	
O2	1.3588 (5)	0.0900 (4)	0.3093 (9)	0.0535 (17)	
C1	0.6903 (8)	0.1273 (5)	−0.0276 (12)	0.042 (2)	
C2	0.7578 (8)	0.1655 (5)	−0.1526 (11)	0.0408 (19)	
C3	0.6965 (9)	0.1916 (5)	−0.2956 (12)	0.045 (2)	
H3	0.7402	0.2196	−0.3746	0.054*	
C4	0.5723 (9)	0.1763 (6)	−0.3203 (13)	0.055 (3)	
H4	0.5337	0.1924	−0.4171	0.066*	
C5	0.5052 (10)	0.1369 (7)	−0.2008 (14)	0.062 (3)	
H5	0.4218	0.1262	−0.2180	0.075*	
C6	0.5630 (9)	0.1130 (6)	−0.0536 (14)	0.055 (3)	
H6	0.5170	0.0877	0.0272	0.066*	
C7	0.7411 (7)	0.1089 (5)	0.1327 (11)	0.0368 (18)	
C8	0.6584 (9)	0.0816 (7)	0.2725 (13)	0.060 (3)	
H8A	0.7001	0.0418	0.3345	0.090*	
H8B	0.5829	0.0609	0.2279	0.090*	
H8C	0.6398	0.1248	0.3436	0.090*	
C9	0.9094 (8)	0.1095 (6)	0.3272 (11)	0.046 (2)	
H9A	0.8534	0.1327	0.4068	0.055*	
H9B	0.9184	0.0546	0.3532	0.055*	
C10	1.0320 (8)	0.1490 (5)	0.3342 (10)	0.041 (2)	
H10A	1.0717	0.1369	0.4385	0.050*	
H10B	1.0199	0.2050	0.3292	0.050*	
C11	1.2203 (9)	0.1793 (5)	0.1721 (12)	0.049 (2)	
H11A	1.1904	0.2326	0.1744	0.058*	
H11B	1.2586	0.1701	0.0657	0.058*	
C12	1.3139 (9)	0.1672 (7)	0.3076 (14)	0.059 (3)	
H12A	1.3826	0.2029	0.2920	0.070*	
H12B	1.2760	0.1789	0.4132	0.070*	
C13	1.2577 (9)	0.0373 (6)	0.3351 (11)	0.048 (2)	
H13A	1.2214	0.0473	0.4423	0.057*	
H13B	1.2888	−0.0158	0.3352	0.057*	
C14	1.1597 (8)	0.0450 (5)	0.2050 (10)	0.0362 (18)	
H14A	1.1934	0.0298	0.0990	0.043*	
H14B	1.0914	0.0102	0.2304	0.043*	

### Atomic displacement parameters (Å^2^)

**Table:**

	*U*^11^	*U*^22^	*U*^33^	*U*^12^	*U*^13^	*U*^23^
Cu1	0.0325 (5)	0.0353 (5)	0.0264 (5)	−0.0002 (4)	−0.0019 (4)	0.0039 (4)
Br1	0.0548 (6)	0.0576 (6)	0.0322 (5)	−0.0018 (4)	0.0064 (4)	0.0000 (4)
N1	0.038 (4)	0.044 (4)	0.027 (3)	0.008 (3)	−0.006 (3)	0.008 (3)
N2	0.026 (3)	0.038 (4)	0.017 (3)	−0.008 (3)	0.006 (2)	−0.010 (2)
O1	0.039 (3)	0.060 (4)	0.055 (4)	−0.004 (3)	−0.009 (3)	0.030 (3)
O2	0.028 (3)	0.068 (5)	0.064 (4)	0.005 (3)	0.003 (3)	0.014 (4)
C1	0.039 (5)	0.029 (4)	0.058 (6)	−0.002 (3)	−0.001 (4)	0.009 (4)
C2	0.046 (5)	0.036 (5)	0.040 (5)	0.006 (4)	−0.005 (4)	−0.004 (4)
C3	0.049 (5)	0.038 (5)	0.048 (5)	0.006 (4)	−0.008 (4)	0.001 (4)
C4	0.046 (6)	0.058 (6)	0.061 (6)	0.013 (5)	−0.024 (5)	−0.003 (5)
C5	0.052 (6)	0.068 (7)	0.068 (7)	−0.002 (5)	−0.024 (5)	−0.012 (6)
C6	0.039 (5)	0.058 (6)	0.068 (7)	0.005 (4)	−0.012 (5)	−0.007 (5)
C7	0.029 (4)	0.035 (4)	0.046 (5)	0.006 (3)	0.006 (3)	−0.005 (4)
C8	0.036 (5)	0.091 (8)	0.054 (6)	0.013 (5)	0.003 (4)	0.018 (6)
C9	0.038 (5)	0.065 (6)	0.035 (5)	0.005 (4)	0.011 (4)	−0.001 (4)
C10	0.043 (5)	0.052 (5)	0.029 (4)	0.007 (4)	0.002 (3)	−0.006 (4)
C11	0.061 (6)	0.034 (5)	0.051 (6)	−0.004 (4)	−0.012 (5)	0.003 (4)
C12	0.041 (5)	0.069 (7)	0.066 (7)	−0.014 (5)	−0.011 (5)	−0.009 (6)
C13	0.053 (6)	0.049 (6)	0.041 (5)	0.014 (4)	−0.004 (4)	0.008 (4)
C14	0.045 (5)	0.030 (4)	0.035 (4)	0.002 (3)	0.003 (4)	−0.001 (3)

### Geometric parameters (Å, °)

**Table:**

Cu1—O1	1.877 (6)	C5—H5	0.9300
Cu1—N1	1.917 (7)	C6—H6	0.9300
Cu1—N2	2.095 (6)	C7—C8	1.518 (12)
Cu1—Br1	2.4006 (14)	C8—H8A	0.9600
N1—C7	1.306 (11)	C8—H8B	0.9600
N1—C9	1.486 (11)	C8—H8C	0.9600
N2—C14	1.447 (10)	C9—C10	1.489 (13)
N2—C11	1.494 (11)	C9—H9A	0.9700
N2—C10	1.497 (9)	C9—H9B	0.9700
O1—C2	1.306 (11)	C10—H10A	0.9700
O2—C12	1.411 (13)	C10—H10B	0.9700
O2—C13	1.434 (12)	C11—C12	1.508 (13)
C1—C6	1.413 (12)	C11—H11A	0.9700
C1—C2	1.411 (12)	C11—H11B	0.9700
C1—C7	1.445 (12)	C12—H12A	0.9700
C2—C3	1.408 (12)	C12—H12B	0.9700
C3—C4	1.382 (13)	C13—C14	1.500 (12)
C3—H3	0.9300	C13—H13A	0.9700
C4—C5	1.387 (16)	C13—H13B	0.9700
C4—H4	0.9300	C14—H14A	0.9700
C5—C6	1.407 (14)	C14—H14B	0.9700
			
O1—Cu1—N1	91.1 (3)	H8A—C8—H8B	109.5
O1—Cu1—N2	161.7 (3)	C7—C8—H8C	109.5
N1—Cu1—N2	87.5 (2)	H8A—C8—H8C	109.5
O1—Cu1—Br1	92.2 (2)	H8B—C8—H8C	109.5
N1—Cu1—Br1	157.9 (2)	C10—C9—N1	108.0 (7)
N2—Cu1—Br1	95.99 (16)	C10—C9—H9A	110.1
C7—N1—C9	118.9 (7)	N1—C9—H9A	110.1
C7—N1—Cu1	129.3 (6)	C10—C9—H9B	110.1
C9—N1—Cu1	111.5 (5)	N1—C9—H9B	110.1
C14—N2—C11	110.1 (6)	H9A—C9—H9B	108.4
C14—N2—C10	115.3 (6)	C9—C10—N2	112.0 (7)
C11—N2—C10	112.0 (6)	C9—C10—H10A	109.2
C14—N2—Cu1	110.6 (5)	N2—C10—H10A	109.2
C11—N2—Cu1	109.6 (5)	C9—C10—H10B	109.2
C10—N2—Cu1	98.7 (5)	N2—C10—H10B	109.2
C2—O1—Cu1	124.8 (6)	H10A—C10—H10B	107.9
C12—O2—C13	109.3 (7)	N2—C11—C12	109.4 (7)
C6—C1—C2	118.5 (9)	N2—C11—H11A	109.8
C6—C1—C7	117.7 (8)	C12—C11—H11A	109.8
C2—C1—C7	123.4 (8)	N2—C11—H11B	109.8
O1—C2—C3	114.5 (8)	C12—C11—H11B	109.8
O1—C2—C1	125.8 (8)	H11A—C11—H11B	108.2
C3—C2—C1	119.7 (8)	O2—C12—C11	111.5 (8)
C4—C3—C2	121.0 (9)	O2—C12—H12A	109.3
C4—C3—H3	119.5	C11—C12—H12A	109.3
C2—C3—H3	119.5	O2—C12—H12B	109.3
C5—C4—C3	120.0 (9)	C11—C12—H12B	109.3
C5—C4—H4	120.0	H12A—C12—H12B	108.0
C3—C4—H4	120.0	O2—C13—C14	112.3 (7)
C4—C5—C6	120.2 (10)	O2—C13—H13A	109.1
C4—C5—H5	119.9	C14—C13—H13A	109.1
C6—C5—H5	119.9	O2—C13—H13B	109.1
C5—C6—C1	120.5 (10)	C14—C13—H13B	109.1
C5—C6—H6	119.7	H13A—C13—H13B	107.9
C1—C6—H6	119.7	N2—C14—C13	110.9 (7)
N1—C7—C1	118.8 (8)	N2—C14—H14A	109.5
N1—C7—C8	120.2 (8)	C13—C14—H14A	109.5
C1—C7—C8	121.0 (8)	N2—C14—H14B	109.5
C7—C8—H8A	109.5	C13—C14—H14B	109.5
C7—C8—H8B	109.5	H14A—C14—H14B	108.1
